# The Helix Ring Peptide U11 from the Venom of the Ant, *Tetramorium bicarinatum*, Acts as a Putative Pore-Forming Toxin, Not a New Kv1.3 Channel Blocker. Comment on Boy et al. A New Kv1.3 Channel Blocker from the Venom of the Ant *Tetramorium bicarinatum*. *Toxins* 2025, *17*, 379

**DOI:** 10.3390/toxins18010044

**Published:** 2026-01-15

**Authors:** Steve Peigneur, Diogo Tibery, Jan Tytgat

**Affiliations:** 1 Toxicology and Pharmacology, University of Leuven (KU Leuven), Herestraat 49, P.O. Box 922, 3000 Leuven, Belgium; steve.peigneur@kuleuven.be (S.P.); dtibery@gmail.com (D.T.); 2 Faculty of Chemistry, Institute of Biological Chemistry, University of Vienna, Währinger Straße 38, 1090 Vienna, Austria; 3 Laboratory of Neuropharmacology, Department of Physiological Sciences, University of Brasília, Distrito Federal, Brasília 70910-900, Brazil

Boy et al. [1] have published an article describing a new Kv1.3 channel blocker from the venom of the ant *Tetramorium bicarinatum*. Their study focuses on MYRTXA4-Tb11a (also known as U11, hereafter abbreviated as Tb11a), and is a follow up study to their publication two years earlier [2]. In this first publication, they reported that Tb11a is an insect neuroactive helix ring peptide and it was hypothesized that its pharmacology may involve modulation of a potassium conductance.

Intrigued by this hypothesis, we have investigated Tb11a on a wide panel of voltage-gated potassium channels (Kv1.1, Kv1.3, Kv1.4, Kv1.5, Shaker IR, Kv4.2, Kv7.1, Kv10.1 and KQT1). Our results, published last year [3], have not shown any modulatory effect of 1 μM Tb11a on these channels. Instead, 10 μM Tb11a caused a quick and irreversible cytolytic effect on *Xenopus* oocytes, an effect identical to the cytotoxic effect caused by *Apis mellifera* venom. Based on these observations, we have shown experimental evidence that Tb11a can act as a pore-forming peptide, and as a result hereof, we have concluded that the insecticidal and paralytic effects caused by Tb11a may be explained by putative pore formation of the peptide, not by modulation of voltage-gated potassium channels.

In their recent paper, Boy et al. [1] now argue that the presence of the disulfide bridge in Tb11a is essential for its biological activity, since reduction of the peptide disrupts the peptide’s conformation and impairs the dyad which is needed to explain the block of the human Kv1.3 (hKv1.3) they have used as a target. The relevance of the hKv1.3 target to substantiate the authors’ claim that Tb11a is an insect neuroactive peptide [2] is not given, nor is a dose–response curve. Boy et al. [1] have used human cell lines, such as MCF-7, MCF-10A and SH-SY-5Y, to test oxidized Tb11a, whereas they have used HEK cells to evaluate the effect of possibly not 100% reduced Tb11a. 

In their discourse, they believe that we have used the reduced form of Tb11a in our experiments and therefore have not seen activity on the hKv1.3 channel. With this reply, we want to clarify and emphasize that we did use the oxidized form of Tb11a, as certified by the mass profile we have received from the company, where the peptide was synthesized with the two cysteine residues linked and hence is in its oxidized form (Biomatik, Wilmington, DE, USA), as well as by the exemplification of the mass profile doubly checked inhouse (KU Leuven, Belgium). Figure 1 shows the mass profile of Tb11a, which was checked in house, with 4019.14 as [M+H]^+^, exactly the same mass expected for the theorical mass, confirming the oxidized state of the peptide we have used.

Generally speaking, the following two hallmarks of Kv blockers are important to remember: (1) Relevant Kv1.3 blockers display IC-50 values in the pico- and/or low nanomolar range, not micromolar. For instance, the following IC-50 values have been reported for peptides from animal venom origin (for a recent review, see Cheng et al., 2024) [4]: 25 pM (ShK-233), 27 pM (HsTx1[R14A], 69 pM (ShK-186), 91 pM (ImKTx88) and 110 pM (MgTx). As such, we conclude that Tb11a, with an alleged IC-50 value of approximately 1 μM, can hardly be viewed as a ‘promising’ and new Kv1.3 channel blocker. (2) With reference to the much cited dyad theory, the original paper by Dauplais et al. (1997) [5] and follow-up papers (e.g., Srinivasan et al., 2002) [6] emphasize a well-defined distance between the critical lysine and the aromatic residue of 6.6 ± 1.0 Å, which is not met by peptide Ta11a, an analog of Tb11a.

## Conclusions

Our experimental results confirm that the oxidized helix ring peptide Tb11a from the venom of the ant *Tetramorium bicarinatum* acts as a putative pore-forming toxin [3]. As such, we believe that this can explain the insecticidal and paralytic effects on blowflies and honeybees, as reported by Barassé et al. [2], without the need to invoke modulation of a potassium conductance, as claimed by Boy et al. [1].

## Figures and Tables

**Figure 1:**
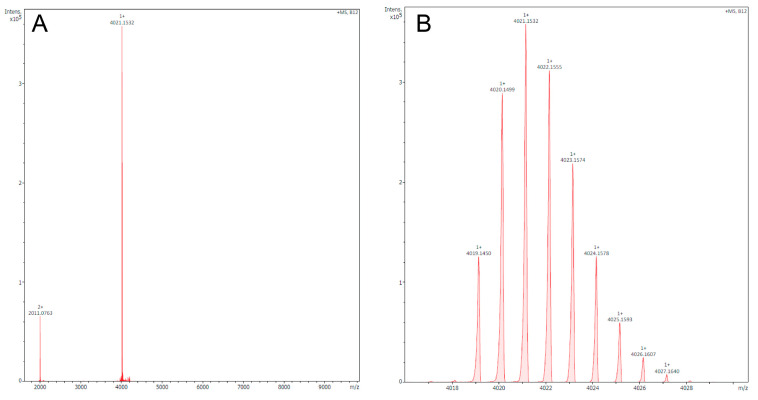
Mass spectrometry profile of Tb11a obtained in a timsTOF Flex MALDI ToF in positive mode. (**A**) Mass spectrum showing all stages of charge labeled with the most abundant peaks. (**B**) Isotopic pattern [M+H]^+^ of Tb11a demonstrating the monoisotopic charged mass of oxidized Tb11a 4019.14 as [M+H]^+^. Mass spectrometry profile of Tb11a obtained in house (KU Leuven, Belgium).

## References

[1] Boy G., Jouvensal L., Téné N., Carayon J.-L., Bonnafé E., Paquet F., Treilhou M., Loth K., Billet A. (2025). A New Kv1.3 Channel Blocker from the Venom of the Ant *Tetramorium bicarinatum*. Toxins.

[2] Barassé V., Jouvensal L., Boy G., Billet A., Ascoet S., Lefranc B., Leprince J., Dejean A., Lacotte V., Rahioui I. (2023). Discovery of an Insect Neuroactive Helix Ring Peptide from Ant Venom. Toxins.

[3] Peigneur S., Tibery D., Tytgat J. (2024). The Helix Ring Peptide U11 from the Venom of the Ant, *Tetramorium bicarinatum*, Acts as a Putative Pore-Forming Toxin. Membranes.

[4] Cheng S., Jiang D., Lan X., Liu K., Fan C. (2024). Voltage-gated potassium channel 1.3: A promising molecular target in multiple disease therapy. Biomed. Pharmacother..

[5] Dauplais M., Lecoq A., Song J., Cotton J., Jamin N., Gilquin B., Roumestand C., Vita C., de Medeiros C.L.C., Rowan E.G. (1997). On the convergent evolution of animal toxins. J. Biol. Chem..

[6] Srinivasan K.N., Sivaraja V., Huys I., Sasaki T., Cheng B., Kumar T.K.S., Sato K., Tytgat J., Yu C., San C.C. (2002). κ-Hefutoxin1, a Novel Toxin from the Scorpion *Heterometrus fulvipes* with unique structure and Function. J. Biol. Chem..

